# Behavioral and Emotional Problems of Prisoners’ Children Based on the Children’s Self-Report

**DOI:** 10.3390/ijerph19010561

**Published:** 2022-01-05

**Authors:** Aneta Domżalska, Bartłomiej Skowroński, Tomasz Wolańczyk

**Affiliations:** 1 Faculty of Applied Social Sciences and Resocialization, University of Warsaw, Krakowskie Przedmieście 26/28, 00-927 Warszawa, Poland; b.skowronski@uw.edu.pl; 2 Department of Child Psychiatry, Medical University of Warsaw, Żwirki i Wigury 61, 02-091 Warszawa, Poland; twolanczyk@wum.edu.pl

**Keywords:** behavioral and emotional problems, prisoners’ children, parental incarceration

## Abstract

The aim of the analyses was to investigate the relations between parental incarceration and the levels of behavioral and emotional problems in children of fathers serving prison sentences, based on the children’s self-report. We tested a criterion group and two control groups. The criterion group consisted of children whose fathers were in prison. The children in control group I were from complete families; the level of problem behaviors in these families and the level of psychological resiliency in these children were similar to the respective levels in the criterion group. Finally, control group II consisted of children whose fathers were not in prison; problem behaviors in their families were basically absent or slight, and their level of resiliency was significantly higher compared to prisoners’ children and control group I. Prisoners’ children exhibited a higher level of emotional and behavioral problems than children from families in which the father was not serving a prison sentence and in which the level of dysfunctions was low. As regards prisoners’ children compared to their peers with a similar level of resiliency and a similar level of problem behavior in the family, statistically significant differences were found only in a few categories of emotional and behavioral problems. Parental incarceration proved to be an additional factor increasing the level of behavioral and emotional problems in children and adolescents—particularly girls—whose fathers were imprisoned.

## 1. Introduction

In the twenty-first century, mental disorders have become a serious social problem, affecting not only adults but also young children and adolescents [1]. The risk of abnormal mental development seems to be even higher in the group of prisoners’ children. On the one hand, abnormalities arise from the experience of parental incarceration; on the other, in many families of these children problem behaviors occur even before the parent’s arrest [2,3]. Scholars therefore point out that prisoner’s children are in a high-risk group for antisocial behavior and emotional or social difficulties [4,5,6].

Children and adolescents function in various social environments on an everyday basis, the key ones being family, school, and peer group [7,8,9]. Human development focuses on the progressive accommodation, throughout the lifetime, between the growing human organism and the changing environments in which it actually grows [8,9]. Moreover, human development is closely related to learning in a social context [7]. This process is characterized by a dynamic and reciprocal interaction of the person, the environment, and behavior [7], which is of particular importance in the context of the functioning of imprisoned people’s children. Self-report surveys are valuable insofar as they provide the diagnostician with results that concern the subject’s functioning in all of these social environments [10,11].

Mental problems in prisoners’ children have been the object of numerous analyses, many of them based on self-report data. Davis and Shlafer [12] examined children whose parents were serving a prison sentence at the time of the study, children of former prisoners, and children whose parents had not been imprisoned. The results showed that children of current and former prisoners scored lower on all indicators of mental health (i.e., internalizing symptoms, suicidal thoughts and suicide attempts, acts of self-mutilation, mental disorders, emotional and behavioral problems, and the treatment of psychological problems) than children whose parents had no prison record, and that children of parents who were in prison at the time of the study were more exposed to mental health risks compared to those whose parents had been imprisoned in the past. Together with other researchers, Davis and Shlafer [13] also examined three groups of subjects (children of parents who were in prison at the time of the study, children of former prisoners, and children of parents with no prison record). They investigated the level of externalizing behavior on the basis of children’s self-report. The results led to the conclusion that children and adolescents from families in which a parent was in prison or had been in prison in the past showed a higher level of behaviors such as aggression, driving under the influence of psychoactive substances, or delinquent behavior than children of parents with no prison record. Moreover, children of currently imprisoned parents reported a considerably higher level of externalizing behavior than those of formerly imprisoned ones.

The increased level of externalizing behavior in prisoners’ children revealed by self-report studies was also noted by other authors [2,14,15,16]. Prisoners’ children showed higher levels of delinquent behavior [2,15,16], aggression [14] and psychoactive substance use [9,10]. Additionally, Kinner and colleagues [14] found a higher level of externalizing behavior in boys. Several studies also confirmed that parental incarceration was related to children’s internalizing problems–depression [14,15], anxiety [14,17]–and to social stigma [18].

It seems that the fact of parental incarceration itself cannot be the main cause behind the emergence and level of problems in prisoners’ children, as not all children of incarcerated parents exhibit behavioral or emotional problems. On the other hand, in many families of this group of children, family difficulties occurred even before parental arrest [2,3]. Perhaps parental incarceration is a factor that intensifies problems. There is evidence that the presence of problems is determined both by harmful upbringing styles [19,20,21] and by individual resources, including low self-worth, low self-efficacy, low sense of coherence, and the absence of abilities such as gentle disposition, coping with stress and difficult emotions, good interpersonal relations, and optimism [22].

Studies on mental problems in children have also revealed gender differences. Girls had significantly higher levels of the following categories of mental problems than boys: withdrawn, somatic complaints, anxious/depressed, and total behavior problems [23]. Boys, in contrast, had higher levels of delinquent behavior and externalizing behavior problems than girls [23]. Similar results were observed in other populations: American [24], Swedish [25], and Norwegian [26]. Unfortunately, we have not found any results of studies conducted among prisoners’ children.

The presence and level of behavioral and emotional problems in prisoners’ children is determined by individual resources and environmental factors. In the present article, we focus on the issues of parental incarceration in the etiology of behavioral and emotional problems. It can be expected that, apart from harmful upbringing styles and the inadequate individual resources of children and adolescents, parental incarceration itself will also be a factor intensifying such problems.

We formulated the following hypotheses:

**Hypotheses** **1** **(H1).** *The level of behavioral problems in prisoners’ children measured using a self-report questionnaire is significantly higher than in their peers from complete families for all categories of problems.*

**Hypotheses** **2** **(H2).** *Boys show a higher level of externalizing behavior problems compared to girls, while girls show a higher level of internalizing behavior problems than boys.*

Definitions:

Prisoners’ children–children and adolescents from 12 to 18 years of age whose fathers were incarcerated in penal institutions at the time of the study;

Prisoner–a father serving a prison sentence at the time of the study;

Complete family–a family in which both the mother and the father look after the child on a daily basis;

Dysfunction is a short term for dysfunctional/problem behavior in the family.

Dysfunctional/problem behavior in the family (selected based on assessment by competent judges) includes, for instance: parents’ drug or alcohol addiction; parents’ use of punishments such as insults, ridicule, reproaches, threats, beatings, or screaming; quarrels between the child and the parent; lack of parental verbal praise towards the child; parent not saying “I love you” to the child; parent not hugging the child; the child witnessing aggression between parents (quarrels, beatings, insults, ridicule, or threats).

## 2. Materials and Methods

The aim of the analyses was to investigate the relations between parental incarceration and the levels of behavioral and emotional problems in children of fathers serving prison sentences, based on the children’s self-report. We defined two research problems:

P1. What are the differences in the levels of behavioral and emotional problems between (1) prisoners’ children, (2) children from complete families with similar levels of resiliency and dysfunctional/problem behavior in the family, and (3) their peers from complete families without dysfunctions?

P2. What are the gender differences in the levels of specific categories of externalizing and internalizing behavior problems in each group?

In the study we measured the following variables: behavioral and emotional problems, resiliency, dysfunctional/problem behavior in the family, and demographic variables.

Behavioral and Emotional Problems. To measure behavioral and emotional problems, we used the version of Achenbach’s questionnaire that is meant to be completed by children and adolescents: namely, the Youth Self-Report (YSR/11–18). The YSR is a measure used to perform a comprehensive assessment of problems in the functioning of children and adolescents that underlie social maladjustment, with various pathomechanisms and symptoms. The author of the Polish adaptation of this questionnaire is Wolańczyk [23]. Using the version for adolescents, administered in the present study, young people aged 11 to 18 assess their own emotional and behavioral competencies and problems [23]. The questionnaire measures the following categories of behavioral and emotional problems: withdrawn, somatic complaints, anxious/depressed, social problems, thought problems, attention problems, delinquent behavior, aggressive behavior, internalizing behavior problems, externalizing behavior problems, and total behavior problems. The reliability of the YSR scales was assessed using their internal consistency. The values of Cronbach’s alpha coefficient ranged from α = 0.62 for Social Problems to 0.95 for total behavior problems score, exceeding 0.70 for seven scales. Additionally, the correlations between all problem scale scores and total behavior problems score were found to be statistically significant in all Achenbach’s measures (i.e., YSR, CBCL, TRF) [23]. The YSR scores proved to be significantly higher in a clinical group, composed of patients with a psychiatric diagnosis, than in the control group (except the Delinquent Behavior score), which attests to the acceptable criterion validity of the measure used in our study [23]. While a number of studies have examined the mental problems of adolescents with currently or formerly incarcerated parents [12,13,15,16] only a few studies among prisoners’ children have been conducted using the YSR [14].

Resiliency. To measure the subjects’ resiliency, we administered the Resiliency Assessment Scale for Children and Adolescents (SPP-18) by Ogińska-Bulik and Juczyński. The reason for the inclusion of this variable and for the use of this particular measure was the selection of participants for one of the control groups based on the mean resiliency score, which made it possible to control for this variable. Many authors point to resiliency as the factor enabling development in the face of adversities and in difficult situations [27,28,29], which means it can act as a protective factor against problems (sampling is discussed at greater length in a further section of this paper). The SPP-18 measures the level of psychological resiliency in children and adolescents aged 12 to 19; it is a self-report measure. It consists of 18 items rated on a 5-point Likert scale (strongly disagree, moderately disagree, hard to say, moderately agree, strongly agree). Cronbach’s alpha coefficient for the whole scale was 0.82. It was found that the higher the level of resiliency, the higher the person’s tendency to manage stress by means of active coping, planning, and—to a slightly smaller degree—by seeking social support, and the lower the tendency to choose strategies such as behavioral disengagement, self-blame, denial, or turning to religion [22].

Dysfunctional/Problem Behavior in the Family. Behaviors that occur in families and have the features of dysfunctions were measured by means of a survey questionnaire. This variable was the second criterion in the selection of controls (the first one was resiliency). Based on the literature, we built an initial pool of items measuring dysfunctional behaviors present in the family. The selection of these items was not arbitrary. Their full list was presented to five competent judges, who rated to what extent each of the items reflected a dysfunction [30]. We used the method of computing interrater agreement proposed by Lawshe [31] and popularized in Poland by Hornowska [30]. For each item of the questionnaire, we computed the content validity ratio according to the formula provided by Lawhse [31]: CVR = (ne-N/2)/(N/2), where CVR stands for content validity ratio, N is the total number of judges, and ne means the number of judges who rated a given item as “essential” for the test. Lawshe [31] specifies that when there are five competent judges the minimum value of CVR should be 0.99. This criterion was met by 46 items, for which a 3-point rating scale was then established: often, sometimes, or never. Specific items were phrased both in educationally desirable terms (e.g., “My mother hugs me”) and in educationally undesirable ones, relating to categories such as addictions, aggression, violence, etc. (e.g., “I witness arguments between my parents”). The respondents completing the questionnaire were children and adolescents (their parents had consented to their participation in the study).

Demographic variables such as gender and age were determined by means of a survey questionnaire.

### 2.1. Sample

The majority of the fathers (N = 44, 61.1%) were serving their sentence under the regular system, 22 fathers (30.6%) were serving theirs under a program-based system, and 6 (8.3%) were imprisoned under a therapeutic system. The sentence execution systems differ in the scope and manner of interventions targeted at the prisoner. The individual program-based intervention system is aimed particularly at rehabilitation. Programs specify the types of employment and education available to prisoners, their contact, above all, with the family and significant others, their use of free time, and other issues (Article 95 Section 2 of the Executive Penal Code). Of the fathers who took part in the study, only one-third were serving their sentence under this kind of system while at the same time being subjected to intensive correctional rehabilitation by the prison staff. The survey results show that 1 prisoner (1.4%) had spent less than half a year in the penal institution, 10 prisoners (13.9%) had been incarcerated for 7 months to 1 year, and 8 (11.1%) had been imprisoned for 13 months to 2 years. The largest groups of prisoners (22 inmates, 30.6%) had been in prison for 25 months to 4 years, and slightly fewer (21 inmates, 29.2%) had been there for 4 to 7 years; 7 prisoners (9.8%) had been incarcerated for 7 to 15 years, and 3 inmates (4.2%) had been imprisoned for 15 to 25 years. None of the inmates taking part in the study was serving a sentence of 25 years or a life sentence. Almost 60% of the inmates had been in prison for between 2 and 7 years. Nearly 1 father in 3 had been incarcerated for up to 2 years, and 1 in 14 reported having been incarcerated for 7 to 25 years. Moreover, the survey results show that 17 of the inmates (23.6%) were incarcerated for the first time, while 55 (76.4%) were recidivists. Some of the collected data (e.g., the types of crime) were incomplete and were excluded from further analysis (e.g., correlation analysis or tests of differences). The largest number of prisoners (20 inmates, 27.8%) had been sentenced under Article 279 of the Penal Code (burglary), and slightly fewer (19, 26.4%) were imprisoned under Article 280 (armed robbery). A further 11 prisoners (15.3%) had committed an offense under Article 148 of the Penal Code (murder), and 8 inmates (11.1%) were in prison for offenses under Article 286 (fraud, ransom, extortion). In the case of 5 inmates (6.9%) the crime had been committed under Article 207 (mistreatment) and 2 inmates (2.8%) were incarcerated under Articles 209 (evading alimony or maintenance payments) and 177 (road accident). Crimes other than those listed above had been committed by 5 prisoners (6.9%). The analysis of the collected data shows that most respondents committed common criminal offenses, predominantly property crimes. Some of the fathers taking part in our study committed crimes against health and life. All fathers who took part in the study were Polish and were serving their prison sentences in Poland.

### 2.2. Criterion and Control Groups

We tested a criterion group and two control groups. The criterion group were children and adolescents whose fathers were serving prison sentences, brought up in dysfunctional/problem families (N = 72). Control group I were children and adolescents from complete families; in this group, the level of dysfunctions/problem behavior in the family and the level of psychological resiliency in children were similar to the respective levels in the criterion group (N = 76). Control group II were children and adolescents whose fathers were not in prison; dysfunctions in their families were basically absent or their level was very low, and the children’s level of resiliency was significantly higher compared to prisoners’ children and control group I (N = 98).

Means and standard deviations for the age of the children and adolescents who took part in the study are presented in Table 1.

Mean age was the lowest in the case of boys from control group I (M = 14.62, SD = 1.93), and the highest in the case of girls from the same control group (M = 15.36, SD = 1.27). The largest group were girls from families without dysfunctions (N = 67), and the smallest group were boys from such families (N = 31).

The inclusion of two control groups was justified, above all, by the obvious and expected differences in behavioral and emotional problems between prisoners’ children and their peers from families without dysfunctions. After all, as shown by research results [32], one of the factors predisposing children to behavioral problems is family members—especially parents—breaking the law. For this reason, we decided to select one more control group (control group I), which differed from prisoners’ children only in terms of father’s incarceration status. We thus controlled for the variables that could confound the results: children’s resiliency and dysfunctional/problem behavior present in their families.

The research procedure was as follows: we administered all the measures chosen to prisoners’ children. The scores on dysfunctional behavior in the family and the resiliency score (means and standard deviations) served as the criteria for selecting children and adolescents for control group I. The sampling criteria applied in the recruitment of children and adolescents for the groups are presented in Table 2.

The expected differences between the groups in terms of dysfunctional behavior and resiliency were verified by means of the Kruskal–Wallis test, which is a nonparametric counterpart of analysis of variance, and Dunn’s (post hoc) test, as reported in Table 3.

As shown by the data presented in the table, control group I consisted of children from complete families, whose levels of resiliency and problem behavior in the family were close to the respective levels in prisoners’ children. Control group II consisted of children characterized by a significantly lower level of problem behavior in the family and a significantly higher level of resiliency compared to prisoners’ children and to their peers from control group I.

In the case of the criterion group, research was carried out in penal institution (prisons and remand prisons), courts (probation teams), social welfare institutions (social welfare centers, Warsaw Family Support Center), non-governmental organizations helping the socially excluded, and junior and senior high schools.

The research in various penal institution in Poland was carried out after obtaining consent from the directors of these institutions and from the inmates. The study involved those incarcerated fathers who reported that they had children between the ages of 12 and 18. The fathers were also asked to provide the mothers’ telephone number so that the researchers could obtain consent to each child’s participation in the study from the mother as well. Most of the incarcerated fathers did not provide the telephone data, even though other personal data were not requested. If an inmate completed the questionnaire but did not provide the other parent’s or legal guardian’s telephone contact information, the results of that inmate’s questionnaire were not qualified for further research procedure. If the father provided the other parent’s or legal guardian’s telephone number, that person was contacted directly and asked for consent to the child’s participation in the study. At this stage, not all parents or legal guardians were willing to have their child participate in the study, and it was not uncommon for the children themselves to refuse to participate. If the parent or legal guardian did consent, the tests involving the child were carried out at the family’s place of residence.

The reverse research procedure was also applied. With the consent and through the agency of probation officers and employees of social welfare centers, non-governmental organizations, or schools, we contacted the mothers or legal guardians of the children whose fathers were imprisoned. Then, after obtaining their consent to the participation of their children in the study and with the consent of the children themselves, we conducted the research at the families’ places of residence or at the office of the institution or organization through which we contacted the families. Then we received the incarcerated fathers’ contact information from the children’s mothers or legal guardians and contacted the fathers in order to conduct the research. The research took place at penitentiary facilities.

Research involving children and adolescents who constituted the control groups took place in schools and community day care centers. It was conducted among children from complete families (where fathers were not in prison). As in the case of the criterion group, we obtained consent from parents or legal guardians for the children’s participation in the study.

## 3. Results

The presentation of results will begin with the differences in specific categories of behavioral and emotional problems between the three groups of subjects: (1) prisoners’ children, (2) children from complete families with dysfunctions, and (3) children from families without dysfunctions. Next, we will present gender differences within each of these groups.

### 3.1. Between-Group Differences

The analysis of results will begin with the presentation of descriptive statistics, which are shown in Table 4.

The level of behavioral and emotional problems was found to be the highest in the group of girls whose fathers were in penal institutions (M = 60.48, SD = 32.77) and in the group of boys whose fathers were in prisons or remand prisons (M = 53.94, SD = 32.52). In the group of prisoners’ children, girls reported a higher level of problems than boys in the following categories: withdrawn, somatic complaints, anxious/depressed, thought problems, attention problems, delinquent behavior, internalizing behavior problems, and externalizing behavior problems. Boys, by contrast, had higher mean scores on social problems and aggressive behavior. Moreover, prisoners’ children (boys and girls) scored higher on all categories of problems than their peers in the two control groups.

To assess the significance of differences between the subjects, we performed a one-way analysis of variance and the Games–Howell post hoc test, recommended for use in situations involving groups unequal in size and with no homogeneity of variance [33]. The differences between prisoners’ children and controls in terms of specific categories of problems, measured using Achenbach’s Youth Self-Report (YSR), are presented in Table 5.

The level of behavioral and emotional problems in boys whose fathers were in prison was significantly higher than in boys from complete families with a similar level of resiliency and with a similar level of dysfunctional/problem behavior in the family (control group I) in two categories: thought problems and attention problems. Moreover, compared to boys from families in which dysfunctional behaviors were not present or in which their level was very low, prisoners’ sons had a higher level of problems in the following categories: withdrawn, somatic complaints, anxious/depressed, social problems, attention problems, delinquent behavior, aggressive behavior, internalizing problems, externalizing problems, and total behavior problems.

A similar pattern of differences between the three groups: prisoners’ children (1), control group I (2), and control group II (3), emerges among girls. The girls whose fathers were incarcerated in penal institutions had significantly higher scores, indicating higher levels of behavioral and emotional problems, in all specific categories (including both internalizing and externalizing problems) than girls from families with a negligible level of dysfunctional/problem behavior (control group II). Details are presented in Table 6.

Moreover, prisoners’ daughters reported higher levels of attention problems, delinquent behavior, aggressive behavior, externalizing problems, and total behavior problems than girls from complete families with a similar level of dysfunctional/problem behavior (control group I).

Before commencing the study, we predicted that the level of all categories of problems would be significantly higher in prisoners’ children than in their peers from complete families (H1). This hypothesis was supported for most of the categories of behavioral and emotional problems that we measured. This finding is true for both girls and boys.

### 3.2. Gender Differences

In order to assess gender differences, we applied the Mann–Whitney U test. The analyses were performed separately for the groups of (1) prisoners’ children, (2) children from complete families with dysfunctions (control group I), and (3) children from families without dysfunctions (control group II).

In the group of prisoners’ children, significant differences between boys and girls were present in the categories of withdrawn (U = 474.5; *p* < 0.05), anxious/depressed (U = 461; *p* < 0.05), and internalizing problems (U = 441.5; *p* < 0.05). The analysis confirmed that it was girls who exhibited significantly higher levels of these variables. This means they were significantly more withdrawn, depressed, and anxious than boys. In the case of the remaining categories of behavioral and emotional problems, differences between boys and girls turned out not to be statistically significant.

In the case of the remaining two groups (Control group I and Control group II), the only category of problems that differentiates girls from boys is anxious/depressed (U = 507.5; *p* < 0.05 and U = 750.5; *p* < 0.05, respectively). In both cases, it was girls who scored significantly higher on this category of problems. This means they were significantly more anxious and depressed than boys. This finding is true both for children and adolescents from complete families with dysfunctions (control group I) and for their peers from families without dysfunctions (control group II).

Hypothesis H2 was partially supported, as there were no statistically significant differences between boys and girls in externalizing behavior problems, whereas significant differences between girls and boys were found in the level of internalizing problems (e.g., anxiety, depression, withdrawal).

## 4. Discussion

The results of the study confirm that prisoners’ children exhibited higher levels of emotional and behavioral problems than children from families in which the father was not serving a prison sentence and in which the level of dysfunctions was lower than in the criterion group and control group I. This was established on the basis of differences in all categories of problems in the case of girls (withdrawn, somatic complaints, anxious/depressed, social problems, thought problems, attention problems, delinquent behavior, aggressive behavior, internalizing problems, externalizing problems, and total behavior problems) and in nearly all categories of problems in the case of boys, the exception being thought problems.

As regards prisoners’ children compared to their peers with a similar level of resiliency and a similar level of dysfunctional/problem behavior in the family (control group I), statistically significant differences were found only in a few categories of emotional and behavioral problems. Let us remember that these were children from families which differed from the families of prisoners’ children only by the father’s absence. Sons of fathers serving a prison sentence had higher levels of thought problems and attention problems, whereas daughters of incarcerated fathers scored higher on attention problems, delinquent behavior, aggressive behavior, externalizing problems, and total behavior problems. It can therefore be concluded that prisoners’ daughters are more exposed to the negative consequences of father’s absence than sons, with higher levels of problems in five categories, including the total behavior problems score. In this respect, the results of our research correspond with the results of studies conducted by other authors, who reported that, although numerous mediating factors were controlled for, the risk of negative consequences in psychosocial development was still increased among prisoners’ children [4,5,6]. Our results are consistent with those reported by other authors both as regards the higher level of externalizing problems in prisoners’ children [2,14,15,16] and as regards the higher level of internalizing problems [14,15,17,18].

The second hypothesis postulated that boys were characterized by a higher level of externalizing problems compared to girls and that girls had a higher level of internalizing problems compared to boys. This hypothesis was partly supported. We only found statistically significant differences in the category of internalizing problems in girls, but no significant differences in the category of externalizing problems in boys. Daughters of incarcerated fathers scored higher on withdrawn, anxious/depressed, and internalizing problems than prisoners’ sons. Girls from the control groups had a higher level of anxious/depressed problems than boys from the control groups. Our findings are partly consistent with the study by Kinner and colleagues [14], who found that girls were higher in internalizing problems while boys were higher in externalizing ones.

## 5. Conclusions

The present study has shown that the very fact of parental incarceration can be an additional factor increasing the levels of behavioral and emotional problems in children and adolescents whose fathers are imprisoned in penal institutions, particularly in the case of girls.

This means that children and adolescents whose fathers are serving prison sentences belong to risk groups and require support and help. Reaching these families with an offer of assistance remains a problem. For this reason, prisoners’ children are sometimes referred to in the literature as the “invisible victims of the justice system” [34], “forgotten victims” [35], “orphans of justice” [36] and the “unseen victims of the prison boom” [37].

Neither in Poland nor in other European Union countries is there a register of prisoners’ children specifying their number [38,39]. This may stem from the intention to avoid stigmatizing these families. However, the authorities should consider establishing a special department within the prison service that would look after incarcerated people’s families, especially their children. The role of these centers would be to cooperate with governmental organizations subordinate to the Ministry of Justice or to the Ministry of Family, Labor, and Social Policy (e.g., social welfare centers) and with non-governmental organizations, which would offer specialized support programs for these children and families in their place of residence. After all, one can hardly implement aid activities responding to the actual diverse needs of these children and their families if it is almost impossible to reach these families.

It would be beneficial to prepare practical programs with solutions and activities to help children solve their emotional, behavioral, and social problems and cope with the situation. This could be achieved through individual and group psychotherapy or through additional educational and development activities. Such programs should take account of the differences between girls and boys in the frequency and intensity of specific types of emotional and behavioral problems. Daughters of incarcerated fathers scored higher on internalizing problems than boys and require special support in this regard. In order to be effective in helping prisoners’ children, their parents should also be provided with greater psychosocial and pedagogical support. While a convicted parent is subject to various penitentiary interventions, including programs targeted at parents, a parent who is not incarcerated often does not have an opportunity to benefit from programs of this kind. The latter should be especially supported in their functioning as a parent.

The present study is a valuable contribution to research on emotional and behavioral problems in prisoners’ children, and its results highlight selected areas of the functioning of this group.

The novelty of our research consisted of the inclusion of two control groups. One of them (control group II) was composed of children from complete families with a level of dysfunctions lower than in the families of incarcerated fathers, and the other one (control group I) was composed of children from complete families which differed from the families of prisoners’ children only in terms of father’s incarceration status. In this way, we controlled for the variables that, if ignored, may have confounded the results: children’s resiliency and problem or dysfunctional behaviors present in their families. The main limitation of our research was the small sample size. It would be an interesting challenge to conduct a study with a larger one.

The present study is an incentive to undertake further research projects devoted to the functioning of incarcerated parents’ children. It would be interesting to study children whose mothers rather than fathers are serving prison sentences and compare the findings with the results of the current project.

## Figures and Tables

**Table 1:** Age and Gender of Prisoners’ Children and Their Peers from the Control Groups.

Prisoners’ Children				Children from Complete Families with Dysfunctions (Control Group I)				Children from Families without Dysfunctions (Control Group II)			
Girls (N = 35)		Boys (N = 37)		Girls (N = 44)		Boys (N = 32)		Girls (N = 67)		Boys (N = 31)	
M	SD	M	SD	M	SD	M	SD	M	SD	M	SD
15.22	2.35	14.64	2.12	15.36	1.27	14.62	1.93	14.76	1.67	14.77	1.58

**Table 2:** Criterion and Control Group Sampling Scheme.

Criteria	Groups		
	Criterion Group (N = 72)	Control Group I (N = 76)	Control Group II (N = 98)
Family structure	Incomplete family Father in a penitentiary institution	Complete family Father not in a penitentiary institution	Complete family Father not in a penitentiary institution
Dysfunctions in the family	Level similar to that in control group I	Level similar to that in the criterion group	None or a low level
Resiliency	Level similar to that in control group I	Level similar to that in the criterion group	Level of resiliency significantly higher than in the remaining groups

**Table 3:** Differences Between the Groups in Terms of Problem/Dysfunctional Behavior and Resiliency.

	Groups	N	M Rank	Kruskal–Wallis *H*	*df*	*p*	Post Hoc
Resiliency	Prisoners’ children (1)	72	101.06	22.029	2	0.000	1 > 3 2 > 3
	Control group I (2)	76	111.68				
	Control group II (3)	98	149.16				
Problem behavior	Prisoners’ children (1)	72	152.94	127.40	2	0.000	1 > 3 2 > 3
	Control group I (2)	76	175.50				
	Control group II (3)	98	61.55				

**Table 4:** Descriptive Statistics for Specific Categories of Behavioral and Emotional Problems in Prisoners’ Children and Controls, Measured with the Youth Self-Report (YSR).

		Prisoners’ Children				Children from Complete Families with Dysfunctions (Control Group I)				Children from Families without Dysfunctions (Control Group II)			
		Boys		Girls		Boys		Girls		Boys		Girls	
		M	SD	M	SD	M	SD	M	SD	M	SD	M	SD
YSR	Withdrawn	4.11	3.45	5.2	2.51	3.94	2.66	4.09	2.68	2.16	2.28	2.0	2.12
	Somatic Complaints	2.51	2.65	3.74	3.35	2.91	2.4	3.61	2.78	1.13	1.28	1.86	2.03
	Anxious/Depressed	7.56	6.22	10.4	6.23	6.44	5.76	9.07	6.33	2.35	2.68	4.01	4.06
	Social Problems	3.97	3.42	3.74	2.69	3.09	3.15	2.41	2.25	1.71	1.77	1.61	1.72
	Thought Problems	1.92	1.90	2.17	2.36	1.84	2.85	1.91	1.89	0.87	1.31	0.88	1.33
	Attention Problems	8.19	4.27	8.2	3.91	5.69	3.63	5.59	2.89	3.22	2.43	3.18	2.38
	Delinquent Behavior	6.38	4.92	7.31	6.54	4.81	3.18	3.59	2.27	1.84	2.32	1.68	1.59
	Aggressive Behavior	15.11	10.18	14.48	8.92	10.44	5.86	9.11	5.44	4.61	3.95	5.16	3.94
	Internalizing Problems	13.62	9.65	18.43	9.14	12.91	9.14	16.2	9.58	5.51	5.26	7.70	6.67
	Externalizing Problems	21.48	14.45	21.8	14.84	15.25	8.69	12.7	7.11	6.45	5.88	6.85	5.07
	Total Behavior Problems	53.94	32.52	60.48	32.77	43.59	26.75	44.25	22.85	20.61	14.55	22.77	16.31

**Table 5:** Differences Between Boys from Three Groups: Prisoners’ Children (1), Control Group I (2), and Control Group II (3) in Specific Categories of Behavioral and Emotional Problems Based on Their Self-Assessment Performed Using the Youth Self-Report (YSR).

	Groups	*df*	*F*	*p*	Games–Howell Test
Withdrawn	1	2	4.522	0.013	1 > 3 2 > 3
	2	97			
	3	99			
Somatic Complaints	1	2	5.555	0.005	1 > 3 2 > 3
	2	97			
	3	99			
Anxious/Depressed	1	2	9.041	0.000	1 > 3 2 > 3
	2	97			
	3	99			
Social Problems	1	2	5.115	0.008	1 > 3 2 > 3
	2	97			
	3	99			
Thought Problems	1	2	2.462	0.091	1 > 2
	2	97			
	3	99			
Attention Problems	1	2	16.249	0.000	1 > 2 1 > 3 2 > 3
	2	97			
	3	99			
Delinquent Behavior	1	2	12.708	0.000	1 > 3 2 > 3
	2	97			
	3	99			
Aggressive Behavior	1	2	17.131	0.000	1 > 3 2 > 3
	2	97			
	3	99			
Internalizing Problems	1	2	9.319	0.000	1 > 3 2 > 3
	2	97			
	3	99			
Externalizing Problems	1	2	16.998	0.000	1 > 3 2 > 3
	2	97			
	3	99			
Total Behavior Problems	1	2	14.016	0.000	1 > 3 2 > 3
	2	97			
	3	99			

**Table 6:** Differences Between Girls from Three Groups: Prisoners’ Children (1), Control Group I (2), and Control Group II (3) in Specific Categories of Behavioral and Emotional Problems Based on Their Self-Assessment Performed Using the Youth Self-Report (YSR).

	Groups	*df*	*F*	*p*	Games–Howell Test
Withdrawn	1	2	23.135	0.000	1 > 3 2 > 3
	2	143			
	3	145			
Somatic Complaints	1	2	8.600	0.000	1 > 3 2 > 3
	2	143			
	3	145			
Anxious/Depressed	1	2	20.556	0.000	1 > 3 2 > 3
	2	143			
	3	145			
Social Problems	1	2	11.303	0.000	1 > 3
	2	143			
	3	145			
Thought Problems	1	2	7.598	0.001	1 > 3 2 > 3
	2	143			
	3	145			
Attention Problems	1	2	33.929	0.000	1 > 2 1 > 3 2 > 3
	2	143			
	3	145			
Delinquent Behavior	1	2	28.255	0.000	1 > 2 1 > 3 2 > 3
	2	143			
	3	145			
Aggressive Behavior	1	2	28.789	0.000	1 > 2 1 > 3 2 > 3
	2	143			
	3	145			
Internalizing Problems	1	2	24.719	0.000	1 > 3 2 > 3
	2	143			
	3	145			
Externalizing Problems	1	2	32.418	0.000	1 > 2 1 > 3 2 > 3
	2	143			
	3	145			
Total Behavior Problems	1	2	32.634	0.000	1 > 2 1 > 3 2 > 3
	2	143			
	3	145			

## Data Availability

The data presented in this study are available on request from the corresponding author. The data are not publicly available due to privacy.

## References

[1] Pawliczuk W., Kaźmierczak-Mytkowska A., Srebnicki T., Wolańczyk T. (2018). Rozpowszechnienie zaburzeń psychicznych wśród dzieci i młodzieży przebywających w placówkach opiekuńczo-wychowawczych, domach dziecka—Przegląd badań epidemiologicznych. Psychiatr. Pol..

[2] Kjellstrand J.M., Eddy J. (2011). Parental incarceration during childhood, family context, and youth problem behavior across adolescence. J. Offender Rehabil..

[3] Poehlmann J. (2005). Children’s family environments and intellectual outcomes during maternal incarceration. J. Marriage Fam..

[4] Eddy J.M., Poehlmann J. (2010). Children of Incarcerated Parents: A Handbook for Researchers and Practitioners.

[5] Kjellstrand J.M., Eddy J. (2011). Mediators of the effect of parental incarceration on adolescent externalizing behaviors. J. Community Psychol..

[6] Murray J., Farrington D.P., Sekol I. (2012). Children’s antisocial behavior, mental health, drug use, and educational performance after parental incarceration: A systematic review and meta-analysis. Psychol. Bull..

[7] Bandura A. (1986). Social Foundations of Thought and Action: A Social Cognitive Theory.

[8] Bronfenbrenner B. (1977). Toward an experimental ecology of human development. Am. Psychol..

[9] Bronfenbrenner B. (1986). Ecology of the family as a context for human development: Research perspectives. Dev. Psychol..

[10] Paulhus D.L., Vazire S., Robins R.W., Fraley R.C., Krueger R.F. (2007). The self-report method. Handbook of Research Methods in Personality Psychology.

[11] Moskowitz D.S. (1986). Comparison of self-reports, reports by knowledgeable informants, and behavioral observation data. J. Pers..

[12] Davis L., Shlafer R.J. (2017). Mental health of adolescents with currently and formerly incarcerated parents. J. Adolesc..

[13] Ruhland E.L., Davis L., Atella J., Shlafer R.J. (2020). Externalizing behavior among youth with a current or formerly incarcerated parent. Int. J. Offender Ther. Comp. Criminol..

[14] Kinner S.A., Alati R., Najman J.M., Williams G.M. (2007). Do paternal arrest and imprisonment lead to child behavior problems and substance use? A longitudinal analysis. J. Child Psychol. Psychiatry.

[15] Miller H.V., Barnes J.C. (2015). The association between parental incarceration and health, education, and economic outcomes in young adulthood. Am. J. Crim. Justice.

[16] NeMoyer A., Wang Y., Alvarez K., Canino G., Duarte C.S., Bird H., Alegría M. (2019). Parental incarceration during childhood and later delinquent outcomes among Puerto Rican adolescents and young adults in two contexts. Law Hum. Behav..

[17] Bradshaw D., Hannigan A., Greaven A.M., Muldoon O. (2019). Longitudinal associations between parental incarceration and children’s emotional and behavioural development: Results from a population cohort study. Child Care Health.

[18] Johnson E., Arditti J., McGregor C. (2018). Risk, protection, and adjustment among youth with incarcerated and nonresident parents: A mixed-methods study. J. Child Fam. Stud..

[19] Kjellstrand J., Yu G., Eddy J.M., Martinez C.R. (2018). Developmental trajectories of externalizing behavior across adolescence. Crim. Justice Behav..

[20] Slaughter J., Davis J.B., Nagoshi C. (2019). Effects of paternal incarceration on father involvement on child behavior outcomes at middle childhood. J. Child Fam. Stud..

[21] Vera J., Granero R., Ezpeleta L. (2012). Father’s and mother’s perceptions of parenting styles as mediators of the effects of parental psychopathology on antisocial behavior in outpatient children and adolescents. Child Psychiatry Hum. Dev..

[22] Ogińska-Bulik N., Juczyński Z. (2011). Prężność u dzieci i młodzieży, charakterystyka i pomiar—Polska skala SPP 18. Pol. Forum. Psychol..

[23] Wolańczyk T. (2002). Zaburzenia Emocjonalne i Behawioralne u Dzieci i Młodzieży w Polsce.

[24] Achenbach T.M. (1991). Manual for the Youth Self-Report and 1991 Profile.

[25] Broberg A.G., Ekeroth K., Gustafsson P.A., Kansson K., Hägglöf B., Ivarsson T., Larsson B. (2001). Self-reported competencies and problems among Swedish adolescents: A normative study of the YSR. Eur. Child Adolesc. Psychiatry.

[26] Kvernmo S., Heyerdahl S. (1998). Influence of ethnic factors on behavior problems in indigenous Sami and majority Norwegian adolescents. J. Am. Acad. Child Adolesc. Psychiatry.

[27] Davydow D.M., Stewart R., Ritchie K., Chaudieu I. (2010). Resilience and mental health. Clin. Psychol. Rev..

[28] Xu J., Ou L. (2014). Resilience and quality of life among Wenchuan earthquake survivors: The mediating role of social support. Public Health.

[29] Ogińska-Bulik N., Juczyński Z. (2008). Skala Pomiaru Prężności (SPP-25). Now Psychol..

[30] Hornowska E. (2001). Testy Psychologiczne. Teoria i Praktyka.

[31] Lawshe C. (1975). A quantitative approach to content validity. Pers. Psychol..

[32] Wasserman G.A., Miller L.S., Pinner E., Jaramillo B. (1996). Parenting predictors of early conduct problems in urban, high-risk boys. J. Am. Acad. Child Adolesc. Psychiatry.

[33] Field A.P. (2005). Discovering Statistics Using SPSS.

[34] Martynowicz A. (2011). Sytuacja Dzieci, Których Rodzice Odbywają Karę Pozbawienia Wolności.

[35] Matthews J. (1983). Forgotten Victims: How Prison Affects the Family.

[36] Shaw R., Shaw R. (1992). Imprisoned fathers and the orphans of justice. Prisoners’ Children: What Are the Issues?.

[37] Petersilla J., Travis J., Visher C. (2005). From cell to society: Who is returning home?. Prisoner Reentry and Crime in America.

[38] Chojecka J. (2013). Uwięzione dzieciństwo—Bariery procesu resocjalizacji. Stud. Edykacyjne.

[39] Kacprzak A., Warzywoda-Kruszyńska W. (2012). Dzieci osób skazanych na karę pozbawienia wolności. Przegląd badań i analiz problematyki. Bieda Dzieci, Zaniedbanie, Wykluczenie Społeczne.

